# Menopause Delays the Typical Recovery of Pre-Exercise Hepcidin Levels after High-Intensity Interval Running Exercise in Endurance-Trained Women

**DOI:** 10.3390/nu12123866

**Published:** 2020-12-17

**Authors:** Víctor M. Alfaro-Magallanes, Pedro J. Benito, Beatriz Rael, Laura Barba-Moreno, Nuria Romero-Parra, Rocío Cupeiro, Dorine W. Swinkels, Coby M. Laarakkers, Ana B. Peinado, on behalf of the IronFEMME Study Group

**Affiliations:** 1 LFE Research Group, Department of Health and Human Performance, Faculty of Physical Activity and Sport Sciences (INEF), Universidad Politécnica de Madrid (UPM), 28040 Madrid, Spain; vm.alfaro@upm.es (V.M.A.-M.); beanad16@gmail.com (B.R.); laurabarbamoreno91@gmail.com (L.B.-M.); n.romero@upm.es (N.R.-P.); rocio.cupeiro@upm.es (R.C.); anabelen.peinado@upm.es (A.B.P.); 2 Translational Metabolic Laboratory (TML 830), Medical Center, Department of Laboratory Medicine, Radboud University, P.O. Box 9101, 6500 HB Nijmegen, The Netherlands; dorine.swinkels@radboudumc.nl (D.W.S.); Coby.Laarakkers@radboudumc.nl (C.M.L.); 3 Hepcidinanalysis.com, Geert Grooteplein 10 (830), 6525 GA Nijmegen, The Netherlands

**Keywords:** interleukin-6, iron homeostasis, inflammation, endurance exercise, estrogen

## Abstract

Menopause commonly presents the gradual accumulation of iron in the body over the years, which is a risk factor for diseases such as cancer, osteoporosis, or cardiovascular diseases. Running exercise is known to acutely increase hepcidin levels, which reduces iron absorption and recycling. As this fact has not been studied in postmenopausal women, this study investigated the hepcidin response to running exercise in this population. Thirteen endurance-trained postmenopausal women (age: 51.5 ± 3.89 years; height: 161.8 ± 4.9 cm; body mass: 55.9 ± 3.6 kg; body fat: 24.7 ± 4.2%; peak oxygen consumption: 42.4 ± 4.0 mL·min^−1^·kg^−1^) performed a high-intensity interval running protocol, which consisted of 8 × 3 min bouts at 85% of the maximal aerobic speed with 90-s recovery. Blood samples were collected pre-exercise, 0, 3, and 24 h post-exercise. As expected, hepcidin exhibited higher values at 3 h post-exercise (3.69 ± 3.38 nmol/L), but also at 24 h post-exercise (3.25 ± 3.61 nmol/L), in comparison with pre-exercise (1.77 ± 1.74 nmol/L; *p* = 0.023 and *p* = 0.020, respectively) and 0 h post-exercise (2.05 ± 2.00 nmol/L; *p* = 0.021 and *p* = 0.032, respectively) concentrations. These differences were preceded by a significant increment of interleukin-6 at 0 h post-exercise (3.41 ± 1.60 pg/mL) compared to pre-exercise (1.65 ± 0.48 pg/m, *p* = 0.003), 3 h (1.50 ± 0.00 pg/mL, *p* = 0.002) and 24 h post-exercise (1.52 ± 0.07 pg/mL, *p* = 0.001). Hepcidin peaked at 3 h post-exercise as the literature described for premenopausal women but does not seem to be fully recovered to pre-exercise levels within 24 h post-exercise, as it would be expected. This suggests a slower recovery of basal hepcidin levels in postmenopausal women, suggesting interesting applications in order to modify iron homeostasis as appropriate, such as the prevention of iron accumulation or proper timing of iron supplementation.

## 1. Introduction

Iron is an essential element for humans, being necessary for vital processes including oxygen transport and energy production, DNA synthesis, host defense, and cell signaling [1]. Its importance requires well-regulated iron levels in the body, since its lack or excess leads to health problems [2]. The main mechanism for maintaining body iron homeostasis is to balance iron supply with iron utilization and losses [3]. Since iron excretion cannot be controlled, the system is regulated according to the iron demands of the body. Iron supply into the plasma is primarily provided by iron recycling in the macrophages and duodenal absorption in the enterocytes, and if necessary, releasing iron from liver stores as ferritin [1]. The inlet flow of iron to plasma is controlled by hepcidin [4,5,6]. This peptide synthesized in the liver regulates iron release into the circulation by binding and inducing degradation or occlusion of ferroportin (FPN), which is the sole known cellular iron exporter on the cell surface [7,8]. Consequently, FPN is highly expressed by cells and tissues involved in iron transport: duodenal enterocytes, liver Kupffer cells, splenic red pulp macrophages, and periportal hepatocytes [9]. Therefore, hepcidin impairs the release of iron into circulation from absorptive enterocytes and recycling macrophages, reducing its availability. Hepcidin synthesis is enhanced by circulating and tissue iron excess, inflammation and endoplasmic reticulum stress, avoiding iron overload and its availability for pathogenic development. On the contrary, hepcidin downregulation is induced by iron deficiency, erythropoietic drive and hypoxia, sex hormones (testosterone and estradiol), and growth factors, allowing iron mobilization from body stores and duodenal absorption to meet the erythropoietic demands and other tissues needs [3].

Among healthy individuals, women present some special considerations regarding iron homeostasis due to the sex hormone fluctuations experienced during their reproductive age and their stabilization once menopause is reached. The latter is characterized by a permanent drop in estradiol and progesterone levels, and the consequent loss of the menstrual cycle and menstrual bleeding [10]. The decline in estradiol has several effects on women’s physiology, resulting in increased risk of illness and poor quality of life [11]. Curiously, premenopausal and postmenopausal women are commonly at opposite ends of iron homeostasis. While premenopausal women are usually affected by iron deficiency (ID) and iron deficiency anemia (IDA), postmenopausal women commonly begin to accumulate iron, as the losses of this metal noticeably decrease due to the lack of menstrual menses after menopause [12,13]. At the same time, hepcidin concentrations increase with age in women, but not in men, as a consequence of iron accumulation through menopause [14]. The low concentrations of estradiol at this stage of life may also contribute to increase hepcidin as well, since estradiol has been proposed to exert inhibitory signals on hepcidin synthesis that may be weaker during menopause [15,16,17]. However, this progressive increase in hepcidin with age does not seem to counteract completely the gradual accumulation of iron, whose etiology is often unknown. This event may be beneficial in preventing ID and IDA, but also harmful, as iron accumulation has been linked with severe health problems such as DNA damage and cancer, osteoporosis, or cardiovascular diseases, among others [12,18,19,20,21].

Interestingly, endurance exercise is documented to influence hepcidin production and iron homeostasis in both the short and long term [22,23,24,25,26,27,28,29,30]. Regarding the acute effects of exercise, hepcidin increments are observed three hours post-exercise in response to the muscle-derived interleukin-6 (IL-6) surge noticed immediately after endurance exercise [22,24,28,29,31]. In contrast, the chronic effects of exercise on hepcidin are still controversial [23,25,26,27,32,33]. Although exercise and a physically active lifestyle have been proposed to ameliorate or counteract many negative effects of menopause on health [34], the role of exercise in postmenopausal women as a potential tool for dealing with iron accumulation has not been studied in depth. Few studies have investigated this issue, noting a reduction in ferritin stores after Nordic Walking programs in a cohort of postmenopausal elderly women [35,36,37]. However, no differences were found in hepcidin levels before and after the interventions [35,36]. This observation suggests that one of the mechanisms for the reduction in ferritin stores may be the aforementioned transient increments in serum hepcidin after exercise, which may reduce dietary iron absorption in training days [22,38]. However, the acute hepcidin response to exercise has not been described in postmenopausal women, who may experience modifications in intensity or in its usual pattern by chronically low estrogen concentrations. Therefore, this study aims to describe for the first time the hepcidin response to a single interval running exercise session in postmenopausal women, in order to help determine the influence of exercise on iron absorption and its usefulness as a potential tool to prevent iron accumulation within this population.

## 2. Materials and Methods

### 2.1. Participants

Thirteen healthy endurance-trained postmenopausal females were recruited for this study (see Table 1 for participant’s characteristics). To be included in the study, participants were required to meet the following criteria: (i) healthy adult females between 40 and 60 years old reporting a menopausal status for at least a year preceding the study [39]; (ii) not to consume medication that alters vascular function (e.g., tricyclic antidepressants, α-blockers, β-blockers, etc.), or any dietary supplements (including any iron supplementation); (iii); non-smokers; (iv) non-oophorectomized; (v) to perform endurance training between 3 and 12 h per week; (vi) not to present iron deficiency anemia (serum ferritin <20 μg/L, hemoglobin <115 μg/L, and transferrin saturation <16%) [40]. To verify inclusion criteria, the participants provided a self-reported questionnaire and a blood analysis. The Research Ethics Committee of the Universidad Politécnica de Madrid approved the project on 21 December 2015, and participants provided written informed consent. The study was registered on clinicaltrials.gov (ID: NCT04458662).

### 2.2. Study Design

This is an observational controlled study. Participants attended the laboratory on two occasions. On the first visit, they underwent a screening protocol. Firstly, baseline blood samples were collected in a rested and fasted state. A complete blood count, biochemistry, and hormonal analysis were performed to verify the conformity of iron metabolism-related parameters with the inclusion criteria and dismiss any illness, hormonal disorders, or menopause dysfunction. It was followed by a Dual-Energy X-ray Absorptiometry (DEXA) scan to determine body composition variables such us body fat mass (%), total body fat mass (kg), and fat free mass (kg), using a GE Lunar Prodigy apparatus (GE Healthcare, Madison, WI, USA). On the same day, after feeding and resting (a minimum of 2 h after feeding), participants performed a maximal ramp test on a computerized treadmill (H/P/COSMOS 3PW 4.0, H/P/Cosmos Sports & medical gmbh, Nussdorf-Traunstein, Germany) to determine each participant’s peak oxygen consumption ($\dot{V}$O_2peak_) [41]. The laboratory environmental conditions were 708.7 ± 4.9 mmHg, 22.8 ± 1.6 °C and 72.9 ± 6.6% of relative atmospheric humidity. Participants began with a warm-up of 3 min at 6 km/hour. Then, the speed was increased by 0.2 km/hours every 12 s. A slope of 1% was set throughout the test to simulate air resistance. Expired gases were measured breath-by-breath using a gas analyzer Jaeger Oxycon Pro (Erich Jaeger, Viasys Healthcare, Höchberg, Germany). Heart response was continuously monitored with a 12-lead ECG. $\dot{V}$O_2peak_ was determined as the mean of the three highest and continuous 15-s interval $\dot{V}$O_2_ measurements in the incremental test to exhaustion, as previously reported [42]. The maximal aerobic speed (v$\dot{V}$O_2peak_) was recorded as the minimum speed required to elicit $\dot{V}$O_2peak_. For the interval running protocol, the speed equivalent to 85% of the v$\dot{V}$O_2peak_ was calculated. After this screening day, participants reported to the laboratory to perform the interval running protocol.

### 2.3. Interval Running Protocol

Participants attended to the laboratory abstaining from alcohol, caffeine, and any intense physical activity or sport practice the 24 h before. Protocols started between 8 and 10 in the morning to avoid diurnal variability of hepcidin [43] and at least 2 h after participants’ last food intake. Nutritional recommendations were provided to the participants by a nutritionist in order to avoid pro-inflammatory food and dietary supplements (e.g., red meat, processed meat, protein powder, multi-vitamin pills, animal milk and dairy products) 48 h before the protocol [44,45,46]. Environmental conditions of the laboratory were 706.5 ± 4.9 mmHg, 20.7 ± 2.3 °C and 70.2 ± 6.9% of relative atmospheric humidity. Firstly, a blood sample was collected before the running protocol to analyze sex hormones, iron homeostasis parameters, and inflammatory markers. Subsequently, participants started the interval running protocol. This consisted of a 5-min warm up at 60% of the v$\dot{V}$O_2peak_ followed by 8 bouts of 3 min at 85% of the v$\dot{V}$O_2peak_ with 90-s recovery at 30% of the v$\dot{V}$O_2peak_ between bouts. Finally, 5 min cool down was performed at 30% of the v$\dot{V}$O_2peak_. This protocol has been previously reported by Sim et al. [47] to produce sufficient stimulus for hepcidin increments 3 h post-exercise. In addition to the baseline sample, antecubital venous blood samples were collected at three different occasions post-exercise: immediately after (0 h), 3 h, and 24 h after finishing the interval protocol. Participants were allowed to drink water ad libitum between 0 and 3 h post-exercise, but no food was consumed. After 3 h, they followed the aforementioned dietary recommendations until the 24 h post-exercise blood collection.

### 2.4. Blood Collection

All venous blood samples were obtained using a 21-gauge (0.8 × 19 mm, Terumo^®^, Tokyo, Japan) needle. Blood samples for complete blood count were collected in a 3 mL K3E EDTA K3 (Vacuette^®^, Kremsmünster, Austria) and immediately sent to the clinical laboratory of the Spanish National Centre of Sport Medicine (Madrid, Spain) for analysis. Blood samples for serum variables were collected in a 9 mL Z serum separator clot activator tube (Vacuette^®^, Kremsmünster, Austria) and allowed to clot at room temperature for 60 min. Then, they were centrifuged for 10 min at 3000 rpm to obtain the serum (supernatant) and subsequently divided into 600 µL aliquots for storing at −80 °C. When data collection was finished, serum samples were sent to the Spanish National Centre of Sport Medicine (Madrid, Spain) in order to analyze IL-6, tumor necrosis factor alpha (TNF-α), iron, ferritin, transferrin, and C-reactive protein (CRP) in all samples. Additionally, pre-exercise serum samples were also analyzed for 17β-estradiol, progesterone, luteinizing hormone (LH), and follicle-stimulating hormone (FSH). Lastly, duplicate serum samples were sent to the Department of Laboratory Medicine at Radboud University Medical Centre (Hepcidinanalysis.com, Nijmegen, The Netherlands) for the measurement of hepcidin-25 serum concentrations.

### 2.5. Blood Analysis

Samples were allowed to defrost at room temperature and homogenized on a vortex. Serum iron was analyzed by spectrophotometry. On the other hand, ferritin, transferrin, and C-reactive protein were analyzed by turbidimetry. Colorimetry and turbidimetry were conducted using an AU400 clinical analyzer (Beckman Coulter, Brea, CA, USA) and Beckman reagents. IL-6, 17β-estradiol, progesterone, FSH, and LH analysis were conducted using Roche diagnostics GmbH reagents in a Cobas E411 analyzer (Roche diagnostics GmbH, Mannheim, Germany), which uses electrochemiluminescent immunoassay method (ECLIA). TNF-α assays were performed in an Immulite 1000 analyzer (Siemens Healthineers, Malvern, AL, USA), using the methodology of chemiluminescence enzyme immunoassay. Controls were measured after calibration and subsequently at each analysis batch. Lower limit of detection and inter- and intra-assay coefficients of variation (CV) reported by the laboratory can be found in Supplementary Material Table S1.

Serum hepcidin measurements were performed by a combination of weak cation exchange chromatography and time-of-flight mass spectrometry (WCX-TOF MS) using a stable hepcidin-25^+40^ isotope and a secondary reference material [48] as internal standard for quantification [49]. Peptide spectra were generated on a Microflex LT matrix-enhanced laser desorption/ionisation TOF-MS platform (Bruker Daltonics). Hepcidin-25 concentrations were expressed as nmol/L (nM). Reference values can be found at http://www.hepcidinanalysis.com/provided-service/reference-values (accessed on 13th September 2020). All values were determined using secondary reference material for hepcidin assays, whose value is assigned by a primary reference material, allowing traceability to the internationally recognized Système International [48].

### 2.6. Statistical Analysis

Data are expressed as mean and standard error of the mean (±SEM) for figures and mean and standard deviation (±SD) for tables. A Shapiro–Wilk test to assess the normality of the variables was conducted. A one-way ANOVA for repeated measures was used to analyze differences among time of measurement (baseline, 0 h post-exercise, 3 h post-exercise, 24 h post-exercise). Mauchly’s sphericity test was carried out to evaluate whether the sphericity assumption of the variances was violated, in which case the Greenhouse–Geisser correction was applied. Additionally, a Bonferroni post-hoc test was performed to obtain pairwise comparisons. Effect sizes using Cohen’s d and their 95% confidence intervals (CI) were calculated to assess the magnitude of effect on the changes found. Threshold values for Cohen’s d were set as small (≥0.2 and <0.5), moderate (≥0.5 and <0.8), and large (≥0.8) [50]. Statistical significance was set at *p* < 0.05 and all procedures were conducted with SPSS software 25 version (IBM Corp., Armonk, NY, USA).

## 3. Results

Sex hormones baseline levels are shown in Table 1. Two participants exhibited high estradiol levels (>100 pg/mL) and non-depleted ferritin stores (≥30 ng/mL); whose individual response is indicated in the figures. Additionally, figures also show the individual response of participants with depleted ferritin stores (<30 ng/mL) and with non-depleted ferritin stores separately, as the iron status before exercise is considered to influence the hepcidin response to exercise [24]. These groups were not analyzed independently due to the low sample size available.

**Figure 1:**
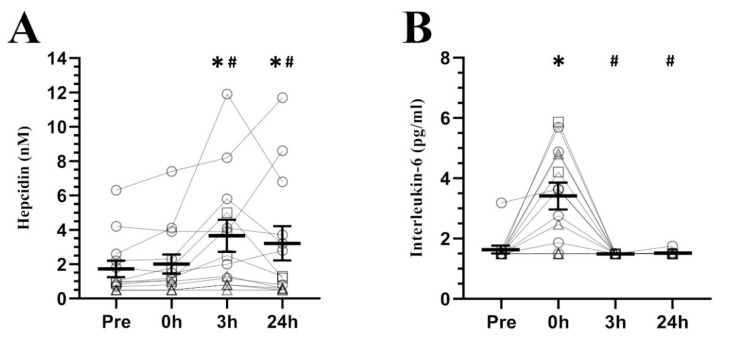
Serum hepcidin (**A**) and interleukin-6 (**B**) concentrations at baseline and its response to a high-intensity interval running session (mean ± SEM). Pre, pre-exercise; 0 h, 0 h post-exercise; 3 h, 3 h post-exercise; 24 h, 24 h post-exercise. * Significantly different from pre-exercise. # Significantly different from 0 h post-exercise. Circles = participants with non-depleted ferritin stores (≥30 ng/mL), Squares = participants with high estrogen concentrations (>100 pg/mL) and non-depleted ferritin stores (≥30 ng/mL), Triangles = participants with depleted ferritin stores (<30 ng/mL).

**Figure 2:**
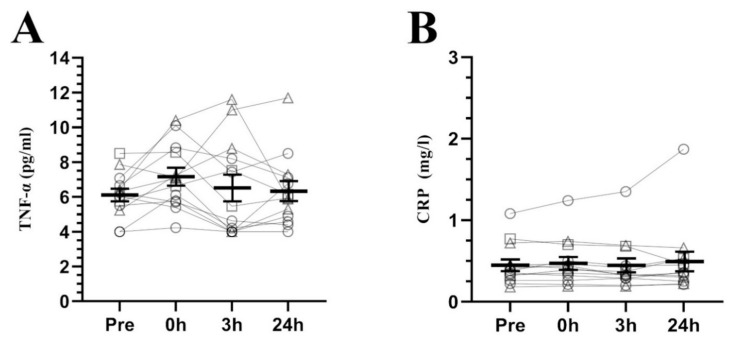
Serum TNF-α (**A**) and CRP (**B**) concentrations at baseline and its response to a high-intensity interval running session (mean ± SEM). Pre, pre-exercise; 0 h, 0 h post-exercise; 3 h, 3 h post-exercise; 24 h, 24 h post-exercise; TNF-α, Tumor necrosis factor alpha; CRP, C-reactive protein. Circles = participants with non-depleted ferritin stores (≥30 ng/mL), Squares = participants with high estrogen concentrations (>100 pg/mL) and non-depleted ferritin stores (≥30 ng/mL), Triangles = participants with depleted ferritin stores (<30 ng/mL).

Regarding hepcidin response to exercise (see Figure 1A), a significant effect of time was found (F_2.23_ = 5.076, *p* = 0.016), showing higher concentrations at 3 h and 24 h post-exercise than pre-exercise (*p* = 0.023, d = 0.72, CI = 0.17 to 1.27; *p* = 0.020, d = 0.52, CI = 0.26 to 0.79, respectively) and 0 h post-exercise (*p* = 0.021, d = 0.59, CI = 0.15 to 1.03; *p* = 0.032, d = 0.41, CI = 0.18 to 0.65, respectively). These differences were preceded by a significant increment of IL-6 (F_1.13_ = 15.587, *p* = 0.001, see Figure 1B) at 0 h post-exercise compared to pre-exercise (*p* = 0.003, d = 1.49, CI = 0.53 to 2.44), 3 h (*p* = 0.002, d = 1.69, CI = 0.68 to 2.70) and 24 h post-exercise (*p* = 0.001, d = 1.67, CI = 0.78 to 2.55). In contrast, the inflammatory markers TNF-α and CRP (see Figure 2A,B, respectively) did not present any differences (F_1.13_ = 0.493, *p* = 0.508; F_3.36_ = 1.070, *p* = 0.374, respectively).

**Figure 3:**
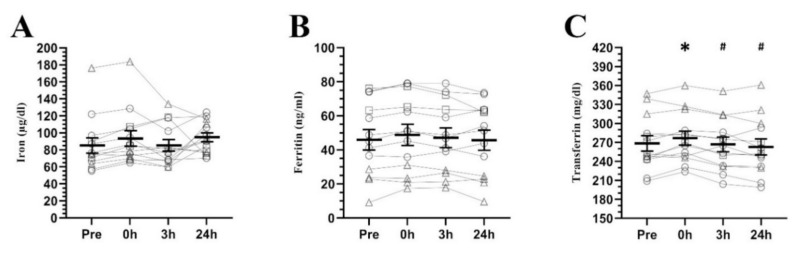
Serum iron (**A**), ferritin (**B**) and transferrin (**C**) concentrations at baseline and its response to a high-intensity interval running session (mean ± SEM). Pre, pre-exercise; 0 h, 0 h post-exercise; 3 h, 3 h post-exercise; 24 h, 24 h post-exercise. * Significantly different from pre-exercise. # Significantly different from 0 h post-exercise. Circles = participants with non-depleted ferritin stores (≥30 ng/mL), Squares = participants with high estrogen concentrations (>100 pg/mL) and non-depleted ferritin stores (≥30 ng/mL), Triangles = participants with depleted ferritin stores (<30 ng/mL).

Regarding iron related parameters (see Figure 3), only transferrin (F_3.36_ = 6.459, *p* = 0.001) showed changes over the measured period of time, finding no differences in iron (F_2.21_ = 1.525, *p* = 0.240) and ferritin levels (F_2.21_ = 2.419, *p* = 0.119). Specifically, transferrin presented higher serum levels at 0 h post-exercise than pre-exercise (*p* = 0.017, d = 0.20, CI = 0.04 to 0.35), 3 h (*p* = 0.003, d = 0.24, CI = 0.08 to 0.40) and 24 h post-exercise (*p* = 0.002, d = 0.32, CI = 0.13 to 0.51).

## 4. Discussion

To the best of our knowledge, this is the first study describing the hepcidin response to interval exercise in postmenopausal females. Hepcidin peaked 3 h after exercise as previously described in men and premenopausal women [22,24,28,29]. However, the main finding is that hepcidin does not seem to fully regain pre-exercise concentrations within 24 h after exercise, suggesting a slower recovery of basal hepcidin levels compared to the recovery reported in men and premenopausal women [22,24,28,29].

IL-6 was elevated just post-exercise as shown in the previous literature, recovering pre-exercise levels at 3 h and 24 h post-exercise [22]. This behavior was coupled with a lack of changes in TNF-α and CRP, indicating that IL-6 acted as a myokine rather than a cytokine, as the data for these variables show no evidence of an inflammatory response [51,52]. In view of this fact, it is surprising that hepcidin levels remained elevated 24 h post-exercise without apparent signs of inflammation.

A factor that may cause this delayed recovery in hepcidin is estradiol. As previously mentioned, estradiol has been reported to exert inhibitory effects on hepcidin synthesis [15,16,17]. Based on this, the low levels of estradiol during the postmenopausal period may hamper the recovery of pre-exercise hepcidin levels at 24 h post-exercise. Accordingly, premenopausal women who performed running trials at moderate intensity during the mid-follicular phase [29] recovered their pre-exercise hepcidin levels at 24 h post-exercise. Although the aforementioned study did not measure sex hormones, estradiol levels are theoretically higher than postmenopausal ones during the mid-follicular phase [53]. This may indicate that estradiol plays a role in the recovery of hepcidin levels after exercise in women. Additionally, other studies that did not take into account the menstrual cycle phase obtained similar results [22]. Curiously, unpublished data from our laboratory showed that premenopausal women who performed the same interval protocol of the present study during their early follicular phase (low estradiol levels) exhibited the highest mean hepcidin concentrations at 24 h post-exercise instead of at 3 h post-exercise. In addition, two of our participants exhibited unusually high estradiol levels (≈100 pg/mL) despite having their last menstruation more than two years ago. Interestingly, they were the only individuals without depleted ferritin stores who presented hepcidin concentrations at 24 h post-exercise similar to those at pre-exercise, while those with depleted ferritin stores (<30 ng/mL) did not show hepcidin response to exercise at any time point (see Figure 1A), as previously shown [24]. These observations are consistent with the hypothesis in which sex hormones may help recover basal hepcidin levels after exercise. However, this explanation needs further and solid research to be confirmed.

On the other hand, the high levels of hepcidin maintained presumably between 3 and 24 h after exercise were not reflected in a reduction of serum iron and/or ferritin levels, at least in the time points measured. Even transferrin showed no change beyond an increase immediately after exercise, which was possibly due to hemoconcentration produced by exercise [54]. These results are consistent with the majority of prior studies showing no acute effect of exercise on ferritin and iron [28,47,55,56], although there are conflicting data with some reports showing elevated [47] or reduced [29,55,56] ferritin and/or iron post-exercise.

The differences between the studies regarding serum iron may be explained in part by the magnitude of the hepcidin response to exercise, but the levels of ferroportin expression in cell membrane may also play a role. Although heme-oxygenase was not measured in the present study, this protein catalyzes the breakdown of heme into iron, and increases in heme activity after exercise have been described [57]. In macrophages, ferroportin is transcriptionally activated by heme released from digested erythrocytes to promote iron recycling [58]. Therefore, an increased expression of ferroportin may occur in the macrophage cell membrane after exercise. This may contribute to maintaining stable serum iron concentrations, and higher levels of hepcidin would be required to significantly reduce circulating iron. Following this hypothesis, increases in hepcidin after exercise would preferentially reduce the absorption of dietary iron, which only represents ≈10% of the plasma iron supply but is key to compensating for daily iron losses [59]. This is only one potential explanation for the lack of differences in plasma iron found in our study and the different results shown in the literature investigating the response of hepcidin to exercise. However, this explanation needs to be experimentally tested. Furthermore, it would be interesting to examine what occurs with both hepcidin and iron in the window of time between 3 and 24 h after exercise in postmenopausal women. This fact would help to clarify whether the peak of hepcidin at 3 h post-exercise decreases the serum iron concentrations in the following hours, and to determine whether the hepcidin concentrations remain elevated throughout the 3 to 24 h post-exercise period.

Regarding baseline hepcidin levels, our participants presented lower concentrations (1.77 nM) than reference values of healthy sedentary women in the same age range whose blood samples were taken, as ours, before 12 pm (4.6 nM) [14]. However, ferritin values were similar or slightly lower than the concentrations observed in healthy sedentary women of the same age [60]. These differences in baseline hepcidin, but not in ferritin, may be indicative of the higher iron demands presented by the sports population [38], since our participants would need lower hepcidin levels and therefore higher iron absorption to maintain average ferritin levels for their age.

## 5. Practical Applications

The present findings may have interesting applications when trying to modify iron homeostasis as appropriate in postmenopausal women. On the one hand, as hepcidin seems to remain elevated from 3 to 24 h, high-intensity interval running exercise may be an interesting tool to reduce iron absorption for almost an entire day in postmenopausal women who present undesired iron accumulation. If repeated over time, this circumstance, along with the ability of exercise to increase iron loses and iron needs for erythropoiesis [61,62], may help reduce ferritin or at least prevent its buildup, as supported by Kortas et al. findings in postmenopausal women after Nordic Walking programs [35,36,37]. However, our exercise protocol was more intense than the Nordic Walking sessions used by the studies of Kostas et al., while the latter were of greater volume. Moreover, it should be noted that our participants were well-trained postmenopausal females and training status may influence iron homeostasis [38]. Therefore, although the hepcidin response to exercise seems to be consistent across different populations, exercise protocols, and modalities [22], it would be useful to confirm this relationship between studies with further research.

Furthermore, it should be noted that the decrease in serum ferritin observed by Kortas et al. [35] was accompanied by a decrease in fasting glucose but not in hepcidin. Given that fasting glucose levels and serum ferritin show a positive correlation [19,35] and that exercise is known to improve insulin resistance [63], there exists the possibility that exercise-induced improvements in the metabolic profile also contribute to a reduction in ferritin stores. The same is true for the inflammatory profile, as exercise also seems to improve this condition [64]. On this basis, exercise may lead to a decrease in serum ferritin by pathways that do not necessarily need the modulation of hepcidin to achieve it. This observation may raise different effects of exercise on ferritin levels in sedentary and endurance-trained postmenopausal women, or even lessen the effects on ferritin if an endurance-trained condition is reached. Moreover, although the postmenopausal state is reached in the late forties or early fifties and lasts until death, it would make sense to find differences between “young” and “older” postmenopausal women facing the same exercise session. Physical fitness and other factors such as inflammation vary with age, so while the present findings are useful, their interpretation should be cautious, as there is still a lack of knowledge between the interaction of exercise, menopause, aging, and iron homeostasis.

On the other hand, the extended period of elevated hepcidin after exercise suggests that postmenopausal females practicing exercise from a non-competitive or health standpoint and suffering from ID would benefit from a schedule alternating training days and days of iron supplementation. It is true that those with low ferritin stores do not seem to experience hepcidin increments after exercise [24]; nonetheless, these individuals may still benefit from this scheme. Once iron depletion is overcome (ferritin ≥30 ng/mL), post-exercise increments of hepcidin are likely to occur, which would hamper the effectiveness of iron supplements, resulting in a delayed recovery of healthy iron stores or, even worse, pushing women back into iron depletion in a vicious circle [38]. Fortunately, providing iron supplements on alternate days and in single doses optimizes iron absorption [65], so the proposed training/iron-supplementation alternance seems to be optimal for maintaining physical activity and its benefits (e.g., 3–4 sessions/week) while improving iron stores in this population.

## 6. Conclusions

This study describes, for the first time, the hepcidin response to high-intensity exercise in endurance-trained postmenopausal women, suggesting a delayed recovery of pre-exercise levels of this hormone based on the high hepcidin levels found 24 h after exercise. However, serum iron was not significantly affected, suggesting more factors than hepcidin affect circulating iron after exercise in postmenopausal women. This warrants further research to clarify the regulation of iron homeostasis in this population.

## Figures and Tables

**Table 1:** Participants’ characteristics and sex hormones concentrations at baseline. Data presented as mean ±SD.

Age (years)	51.5 ± 3.89
Height (cm)	161.8 ± 4.9
Body mass (kg)	55.9 ± 3.6
Body fat (%)	24.7 ± 4.2
$\dot{V}$O_2peak_ (mL/kg/minute)	42.4 ± 4.0
Endurance training experience (years)	7.2 ± 3.1
Training volume during the last 6 months (minute/week)	255 ± 106
17β-estradiol (pg/mL)	28.13 ± 46.95
Progesterone (ng/mL)	0.21 ± 0.13
LH (mIU/mL)	50.32 ± 20.21
FSH (mIU/mL)	90.93 ± 44.60

FSH, follicle-stimulating hormone; LH, luteinizing hormone. $\dot{V}$O_2peak_, peak oxygen consumption.

## Supplementary Materials

The following are available online at https://www.mdpi.com/2072-6643/12/12/3866/s1, Table S1: Lower limit of quantification and coefficients of variation (CV) reported by the laboratory for each of the variables analysed.
