# Supplementary material for: Menopause Delays the Typical Recovery of Pre-Exercise Hepcidin Levels after High-Intensity Interval Running Exercise in Endurance-Trained Women
Source: Nutrients. 2020 Dec 17;12(12):3866. doi: 10.3390/nu12123866 (PMC7766833; doi:10.3390/nu12123866)
Supplement: Supplementary file 1 [file nutrients-12-03866-s001.pdf]

Supplementary Table 1. Lower limit of quantification and coefficients of variation (CV) reported by the laboratory for each of the variables analysed.

Variable	Concentration	Inter-assay CV	Intra-assay CV	Lower limit of detection
**17 β-Estradiol (pg/ml)**	25.4	11.9%	8.5%	5.00
	45.3	6.8%	4.7%	
**Progesterone (ng/ml)**	0.70	23.1%	11.8%	0.05
	9.48	5.2%	2.5%	
**LH (mIU/ml)**	0.54	5.2%	1.8%	0.10
**FSH (mIU/ml)**	1.20	5.3%	1.8%	0.10
**Hepcidin (nM)**	2.7	8.3%	-	0.50
	11.0	4.6%	-	
**Interleukin-6 (pg/ml)**	17.3	8.5%	6.0%	1.50
**TNF-α (pg/ml)**	17.0	6.5%	-	4.00
	34.0	-	3.5%	
**CRP (mg/l)**	0.21	6.4%	4.3%	0.02
**Iron (µg/dl)**	28.3	1.77%	0.66%	1.68
	105.5	1.23%	0.65%	
**Ferritin (ng/ml)**	25.0	3.71%	2.24%	5.47
**Transferrin (mg/ml)**	284.0	0.86%	0.64%	1.00

CRP, C-Reactive Protein; FSH, Follicle-Stimulating Hormone; LH, Luteinizing Hormone; TNF-α, Tumor Necrosis Factor Alpha.
